# Selective photocatalytic hydroxylation and epoxidation reactions by an iron complex using water as the oxygen source†
†Electronic supplementary information (ESI) available: Materials, general instrumentation and additional figures. See DOI: 10.1039/c7sc02780j



**DOI:** 10.1039/c7sc02780j

**Published:** 2017-09-04

**Authors:** Bittu Chandra, Kundan K. Singh, Sayam Sen Gupta

**Affiliations:** a Department of Chemical Sciences , Indian Institute of Science Education and Research Kolkata , Mohanpur , West Bengal , India-741246 . Email: sayam.sengupta@iiserkol.ac.in

## Abstract

The iron complex [(bTAML)Fe^III^–OH_2_]^–^ (**1**) selectively catalyses the photocatalytic hydroxylation and epoxidation reactions of alkanes and alkenes, respectively, using water as the oxygen-atom source. Upon the oxidation of unactivated alkanes, which included several substrates including natural products, hydroxylation was observed mostly at the 3° C–H bonds with 3° : 2° selectivity up to ∼100 : 1. When alkenes were used as the substrates, epoxides were predominantly formed with high yields. In the presence of H_2_^18^O, more than 90% of the ^18^O-labelled oxygen atoms were incorporated into the hydroxylated and epoxide product indicating that water was the primary oxygen source. Mechanistic studies indicate the formation of an active [{(bTAML)Fe^IV^}_2_-μ-oxo]^2–^ (**2**) dimer from the starting complex **1** *via* PCET. The subsequent disproportionation of **2** upon addition of substrate, leading to the formation of Fe^V^(O), renders the high selectivity observed in these reactions.

## Abstract 2

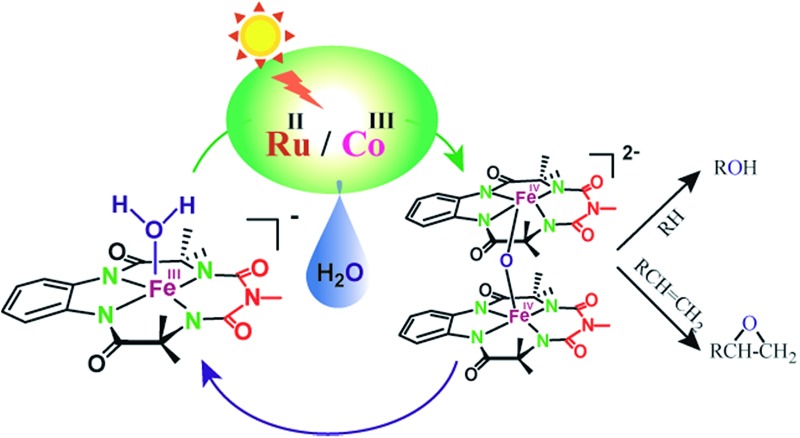
Iron complex catalysed selective and efficient photocatalytic hydroxylation and epoxidation reactions using water as the oxygen atom source has been reported.

## Introduction

The conversion of sunlight to chemical energy has been one of the great challenges for chemists in the quest for a sustainable world.^1^ The inspiration for such a process comes from photosynthesis, wherein sunlight is used to accomplish the energetically uphill water oxidation reaction.^2^ During photosynthesis, a high-valent manganese-oxo cluster has been proposed to be the active intermediate for water oxidation.^3^ Similarly, high-valent iron-oxo complexes have been shown to be the active intermediates for heme/non-heme enzymes and their model complexes which catalyse hydrocarbon oxidation using O_2_ as the oxidant.^4^ Combining these concepts, Inoue *et al.* first demonstrated that visible light can be utilized for the oxidation of organic substrates using water as the oxygen atom source.^5^ Gray *et al.* also developed a photochemical method to generate the intermediates “Compound I” and “Compound II” from peroxidases by using [Ru^II^(bpy)_3_]^2+^ (bpy = 2,2′-bipyridine) as a photosensitizer and [Co^III^(NH_3_)_5_Cl]^2+^ as a mild one-electron oxidant in aqueous medium.^6^ Using these strategies, several photochemical oxygenation systems based on metal complexes (mostly Ru and Mn) as catalysts have been evolved. Examples include various Ru-complexes^7,8^ and Mn-complexes such as Mn–porphyrin,^9^ [(*R*,*R*-BQCN)Mn^II^(OTf)_2_] (BQCN = *N*,*N*′-dimethyl-*N*,*N*′-bis(8-quinolyl) cyclohexanediamine)^10a,b^ and the Mn(v)–nitrido complex, [Mn(N)(CN)_4_]^2–^,^10*c*^ which have been shown to be active catalysts for photocatalytic oxygenation reactions where water was used as the oxygen source. Although ruthenium- and manganese-based metal complexes have been explored for photochemical alcohol, olefin and sulfide oxidation reactions, they have not been shown to catalyse the oxidation of unactivated C–H bonds.^7–10^ The likely reason for this is that the Ru-oxo and Mn-oxo intermediates, formed during these reactions, do not cleave unactivated C–H bonds at rates fast enough for them to function as catalysts for C–H bond oxidation reactions. In contrast, iron-based metalloenzymes (sMMO,^11a,b^ heme and non-heme enzymes^4,11c,d^) and their synthetic models^4b,c,e^ exhibit excellent reactivity and selectivity towards hydrocarbon oxidation. However, for synthetic systems, very few examples are known where a cheap and environmentally benign metal like iron has been used as a catalyst for photochemical oxidation reactions. Recently, the photochemical generation of oxoiron(iv) has been demonstrated using [Fe^II^(N_4_Py)]^2+^ [N_4_Py = *N*,*N*-bis(2-pyridylmethyl)-*N*-bis(2-pyridyl)methylamine]^12^ and [Fe^II^(MePy_2_tacn)]^2+^ [MePy_2_tacn = *N*-methyl-*N*,*N*-bis(2-picolyl)-1,4,7-triazacyclononane].^13^ The complex [Fe^II^(MePy_2_tacn)]^2+^, along with [Ru^II^(bpy)_3_]^2+^ as a photosensitizer and sodium persulfate as a sacrificial oxidant under light irradiation has been shown to catalyse sulfoxidation reactions, albeit with low yields. However, the photochemical hydroxylation of unactivated C–H bonds using the [Fe^II^(MePy_2_tacn)]^2+^ complex described above has not been reported. Mechanistic studies on iron-catalysed hydroxylation reactions have revealed that in both chemical and biological systems, efficient and selective hydroxylation of unactivated C–H bonds has been mostly catalysed by reactive intermediates such as oxoiron(v) (synthetic systems),^14,15,19^ the isoelectronic oxoiron(iv) radical cation (heme enzymes)^4a,11c,d^ and [(μ-O)_2_Fe^IV^_2_] (methane monooxygenase^11a,b^). None of these intermediates have been formed in the photochemical systems reported to date using [Fe^II^(N_4_Py)]^2+^ and [Fe^II^(MePy_2_tacn)]^2+^ complexes, which explains the absence of iron catalysts being used to catalyse photochemical C–H bond oxidation in the literature.

We focused our attention on the peroxidase-mimicking iron(iii) complex of bTAML^14^ (bTAML = biuret-modified tetraamidomacrocyclic ligand) synthesized in our laboratory. The highly electron-donating tetraanionic N-donors are well-known to stabilize high-valent iron-oxo species such as [(bTAML)Fe^V^(O)]^–^ and [(bTAML)Fe^IV^(O)]^2–^ (subsequently referred to as Fe^V^(O) and Fe^IV^(O), respectively).^15^ Moreover, complex **1** in combination with chemical oxidants catalyses the oxidation of unactivated alkanes, alkenes and alcohols selectively.^15b,17b,18,19^ The high selectivity obtained has been attributed to the presence of oxoiron(v) and [{(bTAML)Fe^IV^}_2_(μ-O)]^2–^ (**2**) as active intermediates in these reactions. We have also shown that the oxidant [Ru^III^(bpy)_3_]^3+^, generated either chemically or photochemically, is a competent oxidant to oxidize the [(bTAML)Fe^III^(OH_2_)]^–^ (**1**) complex in solution to the catalytically active [{(bTAML)Fe^IV^}_2_(μ-O)]^2–^ dimer (**2**).^16^ We therefore hypothesized that complex **1**, along with [Ru^II^(bpy)_3_]^2+^ and [Co^III^(NH_3_)_5_Cl]^2+^, can be a competent system to catalyse the photochemical oxidation of synthetically challenging reactions such as the selective hydroxylation of unactivated C–H bonds and epoxidation reactions. The development of such iron-based catalysts would be transformational in the goal to achieve green methods for the selective oxidation of C–H bonds.

**Scheme 1:**
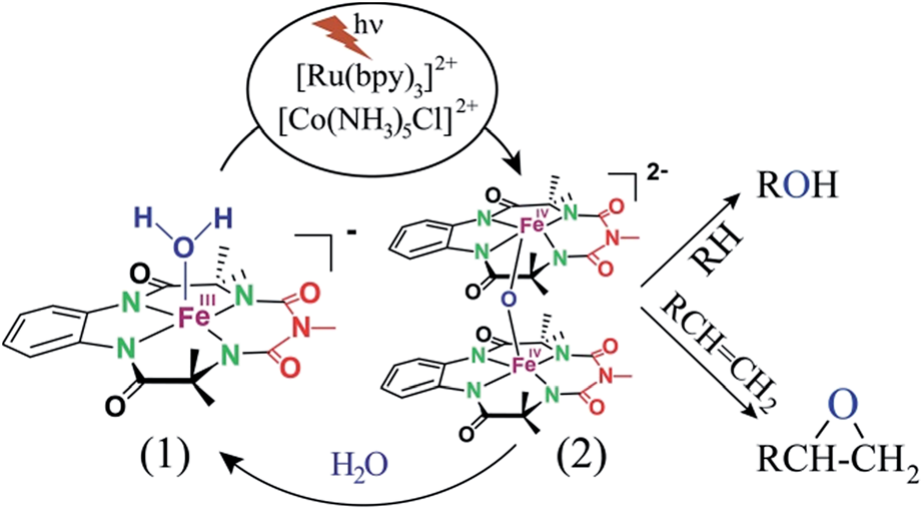
Photocatalytic oxygenation of hydrocarbons.

Herein, we report selective photocatalytic alkane hydroxylation and olefin epoxidation by employing [Et_4_N][(bTAML)Fe^III^(OH_2_)] (**1**) as a catalyst, [Ru^II^(bpy)_3_]Cl_2_ as a photosensitizer, [Co^III^(NH_3_)_5_Cl]Cl_2_ as a mild one-electron acceptor and water as the oxygen atom source. We also demonstrate that under the reaction conditions, intermediate **2** was generated and found to be the active oxidant (Scheme 1). To the best of our knowledge, this represents the first example of the use of an iron-complex to catalyse the photochemical selective oxidation of unactivated C–H bonds and C=C bonds using water as the O-atom donor.

## Results and discussion

### Photocatalytic oxidation of alkanes

**Fig. 1:**
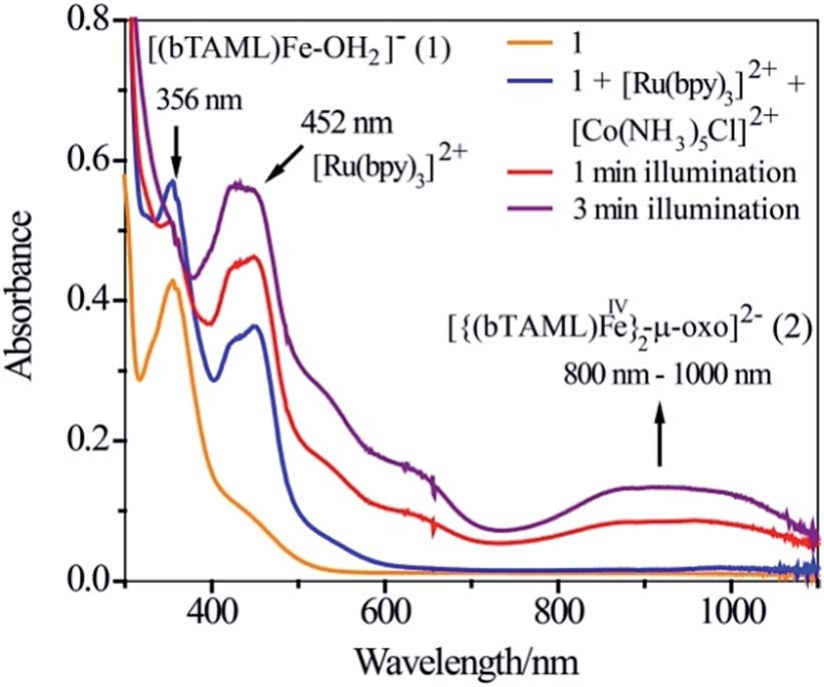
UV-vis spectral scan of a photochemical reaction mixture of **1** (1.0 × 10^–4^ M), [Ru^II^(bpy)_3_]^2+^ (2.0 × 10^–5^ M) and [Co^III^(NH_3_)_5_Cl]^2+^ (6.0 × 10^–4^ M) in acetonitrile-aqueous phosphate buffer (3 : 2 v/v) solvent.

**Fig. 2:**
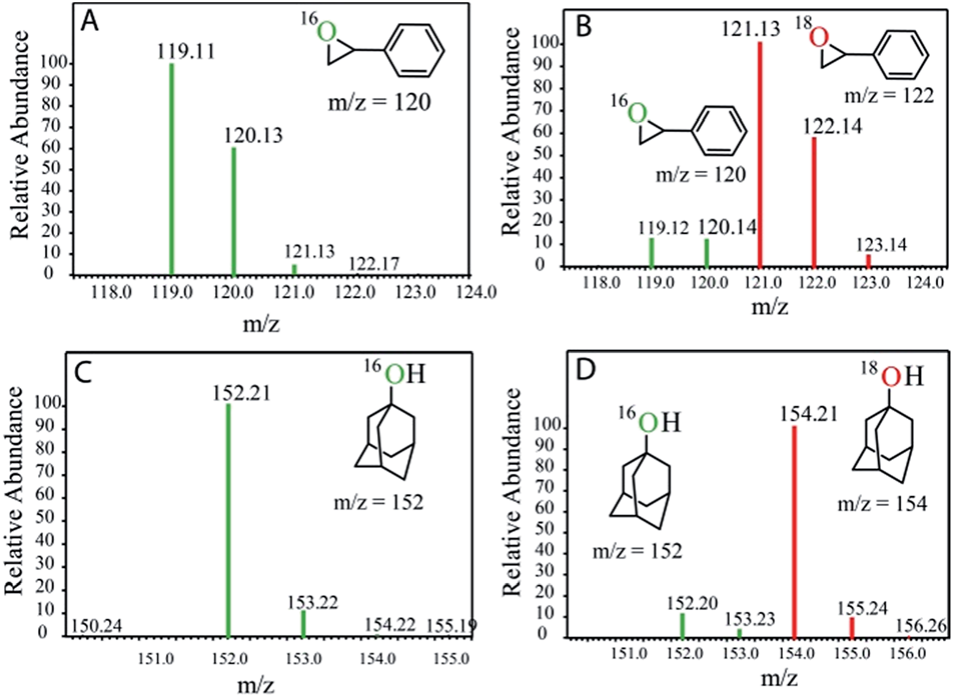
GC-MS spectra of the products after photochemical reaction with (A) styrene in H_2_^16^O, (B) styrene in H_2_^18^O, (C) adamantane in H_2_^16^O, and (D) adamantane in H_2_^18^O.

All of the photocatalytic hydroxylation reactions were performed by employing catalyst **1** (∼3–7 mol% loading), [Ru^II^(bpy)_3_]Cl_2_ and [Co^III^(NH_3_)_5_Cl]Cl_2_ in an acetonitrile–phosphate buffer solution mixture in the presence of light (3 W blue LED, 440 nm) under an argon atmosphere. The photocatalytic hydroxylation of alkanes with different unactivated 3° and activated 2° C–H bonds was attempted under optimized reaction conditions. Adamantane, having twelve 2° and four 3° C–H bonds, was used as a substrate probe to study the regioselectivity using the photocatalytic system. Upon completion of the reaction, 1-adamantanol was formed in good yield (88%) and the 3° : 2° selectivity for adamantane oxidation was calculated to be ∼100 : 1 (Table 1; entry 1). In the case of cumene, which contains six 1° and one benzylic 3° C–H bonds, the 3° hydroxylated product was formed as the major product (67% yield; Table 1, entry 2). Next, *cis*-1,2-dimethylcyclohexane and *cis*-decalin were chosen as the substrates to examine the stereoselectivity of this oxidation. The reaction of *cis*-1,2-dimethylcyclohexane and *cis*-decalin displayed primarily 3° hydroxylated product (99% and 96% yields, respectively) with more than ∼97% retention of configuration under the reaction conditions (Table 1; entries 3 and 4). The high stereo-retention observed excluded the possibility of radical processes since *cis*-1,2-dimethylcyclohexane and *cis*-decalin are known to epimerize to the *trans* isomer if radical processes are operational. In cyclohexane derivatives, the stereochemical orientation of the 3° C–H bonds (axial or equatorial) determines the regioselective outcome of the reaction. For the catalytic hydroxylation of *trans*-decalin, the reaction was comparatively slower with a lower yield (60%) of the oxidized product (Table 1, entry 5) in contrast to that of the *cis* isomer. The oxidation of *trans*-decalin also exhibited oxidation at the methylenic C–H bonds unlike its *cis* congener, resulting in the formation of both alcohol (99% retention of configuration) and ketone at a ratio of 3 : 2. The difference in reactivity between the *cis* and *trans* isomers can be attributed to the strain release in the transition state for the *cis*-isomers.^20^ In short, the hydroxylated products formed after the reaction displayed very high regioselectivity of 3° over 2° C–H bonds (Table 1; entries 3, 4 and 6), where 3° hydroxylated products were formed predominantly. This result is consistent with a similar oxidation reported by us using complex **1** and mCPBA as the oxidant.^19^ Furthermore, the regioselective oxidation of 3° C–H bonds in the natural product derivative of cedrol, cedryl acetate, was performed. Cedrol is a sesquiterpene alcohol found in essential oil, having a very rigid structure with five 3° C–H positions. The substrate was hydroxylated very selectively with good yield, albeit with a low conversion (Table 1, entry 6). In order to find the O-atom source in the product formed, oxidation reactions of *cis*-1,2-dimethylcyclohexane and adamantane were carried out in a mixture of CH_3_CN and H_2_^18^O (3 : 2 v/v). We observed >90% and >95% incorporation of ^18^O-labelled oxygen atoms in 1-adamantanol (Fig. 2D) and (1*S*,2*R* or 1*R*,2*S*)-1,2-dimethylcyclohexanol (Fig. S1†), respectively, which confirmed water as the O-atom source. This also precluded the involvement of an O_2_-based radical pathway during the reaction.

**Table 1:** Photocatalytic hydroxylation of different alkanes by **1** using water as the oxygen source^*a*^

Entry	Substrate	Products	(Other products)	Conversion (%)	Yield^*c*^ (%)
1	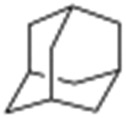	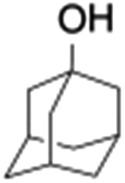	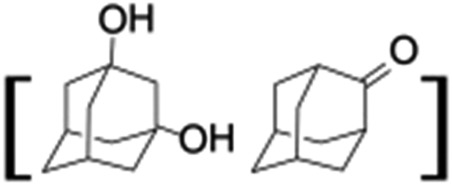	76	88
2	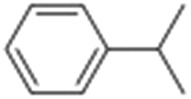	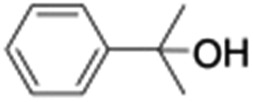	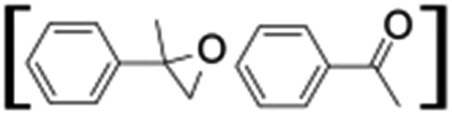	50	67
3	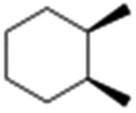	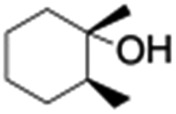	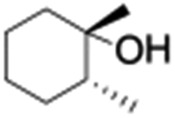	55	99 (60 : 1), (*cis* : *trans*)
4^*b*^	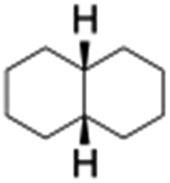	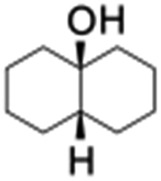	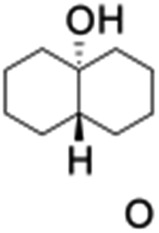	99	96 (65 : 1), (*cis* : *trans*)
5^*b*^	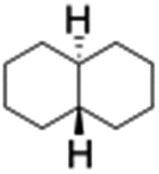	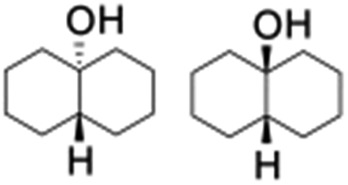	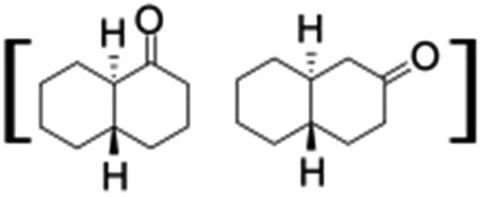	50	60 (20 : 1), (*trans* : *cis*)
6^*b*^	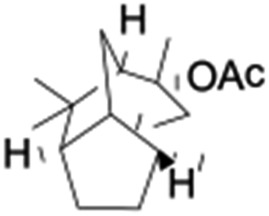	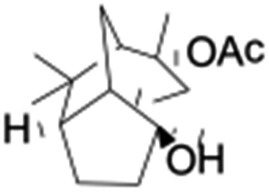		37	97
7	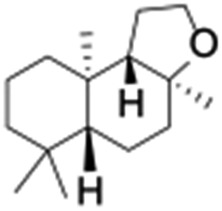	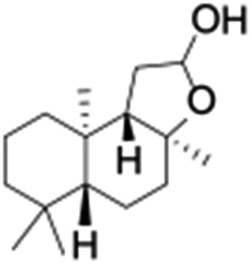	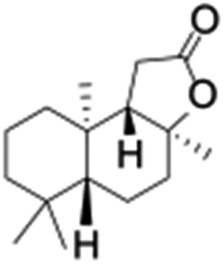	56	98 (7 : 12), (alcohol : ketone)
8	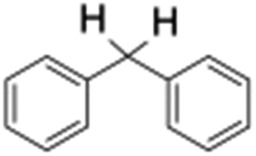	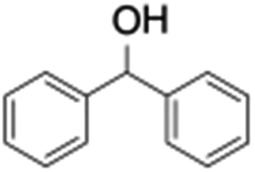	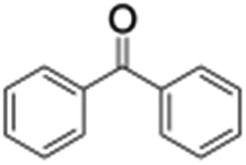	60	94 (7 : 10), (alcohol : ketone)

^*a*^Reaction conditions: **1** (1.0 × 10^–4^ M), [Ru(bpy)_3_]^2+^ (2.0 × 10^–3^ M), [Co(NH_3_)_5_Cl]^2+^ (2.0 × 10^–2^ M) and substrate (3.0 × 10^–3^ M) in acetonitrile-aqueous phosphate buffer (3 : 2 v/v, 10 mM, pH 10). Photoirradiation with LED (3 W, 440 nm), at room temperature (27 °C), under argon for 40 min.

^*b*^Catalyst **1** (2.0 × 10^–4^ M).

^*c*^Yields are based on substrate conversion (the amounts of side products indicated inside the parenthesis are not included); yields and conversions were estimated by GC.

Finally, the selective oxidation of substrates bearing activated methylenic and benzylic C–H bonds was explored. In the case of the substrate ambroxide, oxidation at the α-ethereal C–H bond occurred predominantly among numerous other electronically and sterically accessible secondary and tertiary sites (Table 1; entry 7). At a lower substrate concentration, over-oxidized ketone was the major product formed (Table 1; entries 7 and 8). The alcohol to ketone product ratio increased with increasing substrate concentration (3 mM to 40 mM; Table 2) under the same reaction conditions. Similar results were found when diphenylmethane was employed as the substrate (Table 1; entry 8). This formation of ketone was attributed to the over-oxidation of the hydroxylated product, which was first formed during the hydroxylation reaction (cyclohexanol oxidation has been shown to be 400 times faster than cyclohexane oxidation^18^). The incorporation of ∼80% ^18^O-labelled oxygen atoms in the ketone product of ambroxide (Fig. S2†) also supports this hypothesis. The quantum yields for the photocatalytic oxidation of the alkanes were determined using a standard actinometer (potassium ferrioxalate) and a maximum value of 12.2% was observed in the case of the *cis*-decalin hydroxylation (Table S1†).

**Table 2:** Effect of substrate concentration on the photocatalytic hydroxylation of 2° C–H bonds by **1** using water as the oxygen source^*a*^

Entry	Substrate	Products	Alcohol/ketone (ratio) in different substrate concentrations (mM)			
			3 mM	10 mM	20 mM	40 mM
1	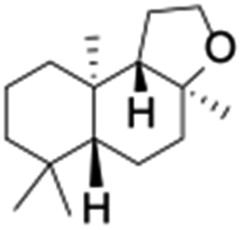	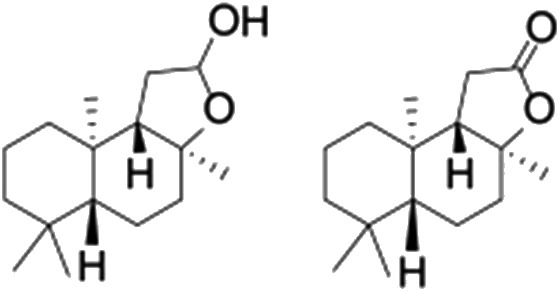	0.6	1.8	2.4	5.3
2	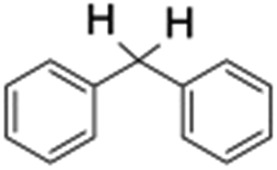	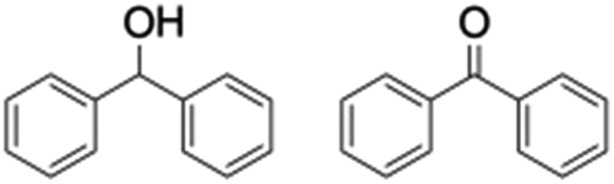	0.7	1.5	3.5	6.1

^*a*^Reaction conditions: **1** (1.0 × 10^–4^ M), [Ru(bpy)_3_]^2+^ (2.0 × 10^–3^ M) and [Co(NH_3_)_5_Cl]^2+^ (2.0 × 10^–2^ M) in acetonitrile-aqueous phosphate buffer (3 : 2 v/v, 10 mM, pH 10). Photoirradiation with LED (3 W, 440 nm), at room temperature (27 °C), under argon for 40 min.

### Photocatalytic oxidation of alkenes

Epoxidation of various alkenes was also performed under optimized reaction conditions by employing catalyst **1** (2 mol% loading) (Fig. S3†). Analysis of the products indicated predominant formation of alkene oxides in moderate to high yields (79–84%) and only a trace amount of the side-product aldehyde (∼5–7% with respect to epoxide) was observed. At first, styrene was chosen as the substrate for the photocatalytic epoxidation reaction where styrene oxide was obtained as the predominant product. Subsequently, different para-substituted styrene derivatives such as 4-chlorostyrene and 4-methoxystyrene were investigated and their corresponding epoxides were obtained with yields of up to 92% (Table 3; entries 1, 2 and 3). The higher amount of epoxide formation for the para-substituted electron-donating group on styrene in comparison to the electron-withdrawing group supports the likely involvement of an electrophilic high-valent iron-oxo intermediate, as has been reported earlier by us.^17*b*^ For *cis*-stilbene, a substrate which contains a sterically constrained double bond, a lower conversion of 58% and a moderate yield of 79% (Table 3; entry 4) was obtained. The *cis*/*trans* product ratio 19 : 2 was estimated by ^1^H-NMR (Fig. S4†) (note: additional stereo-scrambling in the *cis*/*trans* ratio was observed in GC run). The substrate scope was further expanded to include cyclooctene and norbornene (Table 3; entries 5 and 6) where a 94% yield of cyclooctene oxide and a 92% yield of *exo*-norbornene oxide indicate selectivity for the C=C bond over the C–H bonds. For the epoxidation of alkenes, a maximum quantum yield of 18.7% for 4-methoxystyrene was observed (Table S2†).

**Table 3:** Photocatalytic epoxidation of different alkenes by **1** using water as the oxygen source^*a*^

Entry	Substrate	Product	Conversion (%)	Yield (%)
1	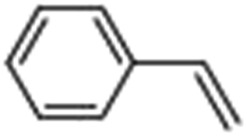	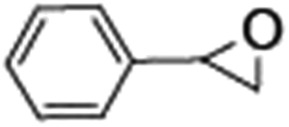	72	90
2	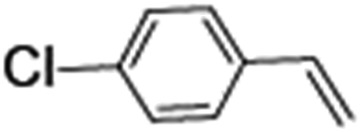	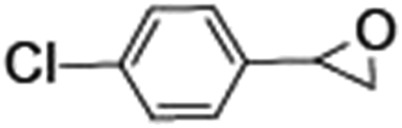	50	84
3	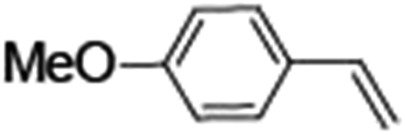	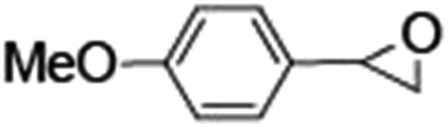	95	92
4	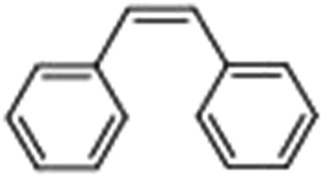	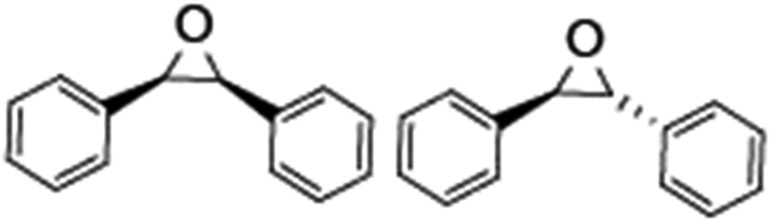	58	79
5	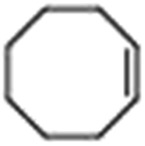	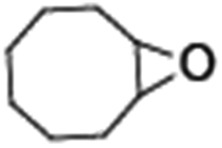	51	94
6	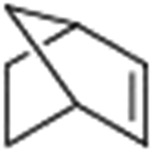	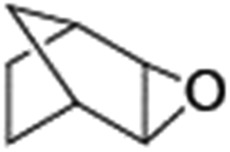	64	92

^*a*^Reaction conditions: **1** (1.0 × 10^–4^ M), [Ru(bpy)_3_]^2+^ (2.0 × 10^–3^ M), [Co(NH_3_)_5_Cl]^2+^ (2.0 × 10^–2^ M) and substrate (5.0 × 10^–3^ M) in acetonitrile-aqueous phosphate buffer (3 : 2 v/v, 10 mM, pH 10). Photoirradiation with LED (3 W, 440 nm), at room temperature (27 °C), under air for 40 min. Yields are based on substrate conversion (the amounts of side products are not included); yields and conversions were estimated by GC.

### Mechanistic insight

Since the operation of free radical oxidation was ruled out, the involvement of a high-valent iron-oxo intermediate during the reaction was investigated. Upon irradiation of the mixture of **1**, Ru^2+^ and Co^3+^ in a 3 : 2 CH_3_CN–phosphate buffer solution mixture with blue LED light (*λ*_max_ = 440 nm), a broad absorption band in the region of 800–1000 nm was observed (Fig. 1; violet coloured spectrum). This new species was assigned as the previously characterized dimer, [{(bTAML)Fe^IV^}_2_(μ-O)]^2–^ (**2**),^15*b*^ which is consistent with the UV-vis spectrum of the chemically synthesized dimer **2** (Fig. S5†). This intermediate species was not observed in the absence of any one of the components (catalyst, Ru^2+^, Co^3+^ or light). Upon addition of the substrate (alkenes or alkanes) to this solution, **2** reacted with the substrate and regenerated the parental complex **1** (Fig. S6†), which restarted the catalytic cycle upon light irradiation with the concomitant formation of the oxygenated product (alcohol or epoxide).

We demonstrated earlier that independently synthesized [Ru^III^(bpy)_3_]^3+^ was competent in oxidizing **1** to form the complex **2**.^16^ We therefore propose that [Ru^III^(bpy)_3_]^3+^, which is generated due to one-electron transfer from the excited state of [Ru^II^(bpy)_3_]^2+^ to the sacrificial oxidant [Co^III^(NH_3_)_5_Cl]^2+^, oxidizes **1** containing an axial H_2_O ligand to generate a putative [(bTAML)Fe^IV^–OH]^–^ species by one-electron and one-proton transfer processes (PCET). This proposition is based on previous electrochemical studies reported by us.^15c,d^ Under neutral or basic conditions (pH 10 in this case), the Fe^IV^–OH species dimerizes immediately to form complex **2** as shown below.
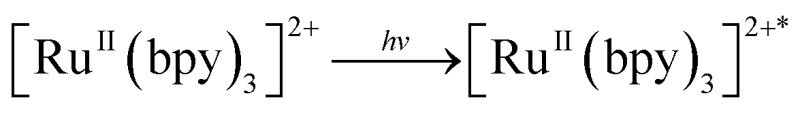
[Ru^II^(bpy)_3_]^2+^∗ + [Co^III^(NH_3_)_5_Cl]^2+^ → [Ru^III^(bpy)_3_]^3+^ + [Co^II^(NH_3_)_5_Cl]^+^[Ru^III^(bpy)_3_]^3+^ + **1** → [(bTAML)Fe^IV^–OH]^–^ + [Ru^II^(bpy)_3_]^2+^[(bTAML)Fe^IV^–OH]^–^ + [(bTAML)Fe^IV^–OH]^–^ → **2**

This is in contrast to the [(N_4_Py)Fe^II^]^2+^ and [(MePy_2_tacn)Fe^II^]^2+^ complexes where the Fe^III^–OH species is oxidized by [Ru^III^(bpy)_3_]^3+^ to form the corresponding Fe^IV^(O).^12,13^ The reactivities of the Fe^IV^ complexes, *i.e.* the Fe^IV^(O) and [(μ-O)Fe^IV^_2_] dimer (**2**), differ. While the high-valent [(N_4_Py)Fe^IV^(O)] and [(Me_2_Pytacn)Fe^IV^(O)] [Me_2_Pytacn = 1-(2-pyridylmethyl)-4,7-dimethyl-1,4,7-triazacyclononane] species are competent in cleaving strong C–H bonds selectively, the low redox potential of the Fe^III^–OH formed prevents the subsequent “rebound” process, thus leading to free radical auto-oxidation.^22^ In contrast, our investigations on the reactivity of the [{(bTAML)Fe^IV^}_2_-μ-oxo]^2–^ (**2**) with alkanes, alkenes and alcohols demonstrate that the dimer exists in equilibrium with the corresponding Fe^V^(O) and Fe^III^ (**1**). Such a proposal is based on our previously reported kinetic and mass spectral studies with **2**.^17*b*^ Upon addition of the substrate, the dimer (**2**) disproportionates into Fe^V^(O) and Fe^III^ (**1**), and the Fe^V^(O) intermediate remains the active oxidant. The primary formation of the *cis*-hydroxylated product (Table 1; entry 3) in reactions with *cis*-1,2-dimethylcyclohexane also supports our reported mechanism that involves C–H bond abstraction by Fe^V^(O) and the subsequent formation of a hydroxylated product by a “rebound” mechanism.^15*b*^ H_2_^18^O labelling experiments with styrene and adamantane result in the formation of more than 90% ^18^O-labelled epoxide and hydroxylated product, respectively, which clearly indicates that water is the primary oxygen atom source (Fig. 2).
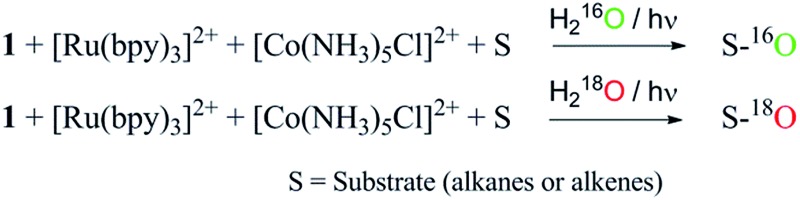


### Proposed photocatalytic reaction cycle

**Scheme 2:**
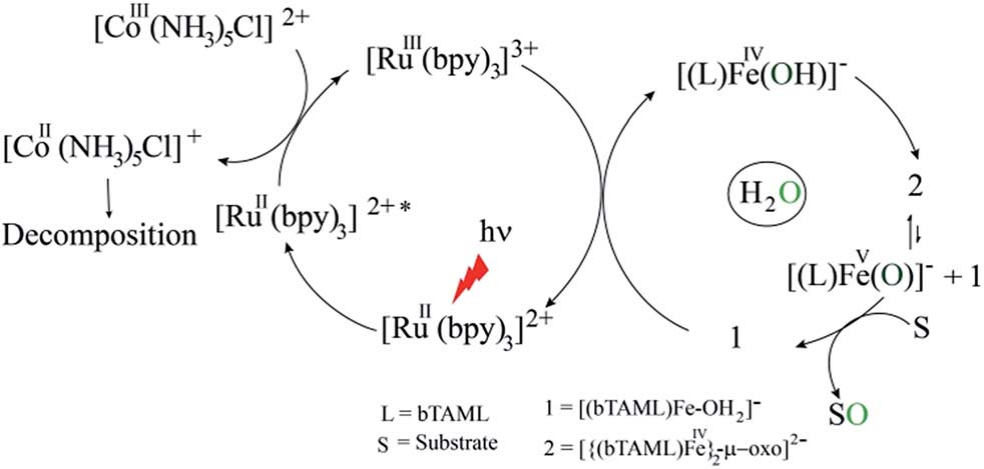
Proposed reaction mechanism.

Finally, on the basis of product analysis, UV-vis spectroscopy, ^18^O-labelling experiments, mass analysis and previously reported observations, we propose the following catalytic cycle for the photochemical oxygenation reaction (Scheme 2). In water, Fe^III^-bTAML (**1**), upon irradiation with light (3 W blue LED, 440 nm) in the presence of a photosensitizer [Ru^II^(bpy)_3_]^2+^ and an electron acceptor [Co^III^(NH_3_)_5_Cl]^2+^, gets oxidized to [(bTAML)Fe^IV^–OH]^–^ which immediately gets converted into dimer **2**. Upon addition of substrate, the dimer disproportionates to Fe^V^(O) and Fe^III^ (**1**). For the hydroxylation reaction, the mechanism likely involves a C–H bond abstraction followed by a rebound process to generate the corresponding alcohol. For epoxidation reactions, the formation of a radical intermediate upon electrophilic attack of the Fe^V^(O) onto the alkene followed by a fast ring closing step is expected.^17*b*^

Before concluding, one important point is worth noting. Irradiation with visible and UV light has been shown to alter the reactivity of intermediates such as [{(corrole)Fe^IV^}_2_-μ-oxo], [Fe^IV^(O)(MePy_2_tacn)]^2+^ and cofacial bis-porphyrin-diiron(iii)-μ-oxo complexes as has been reported by Newcomb *et al.*,^21*b*^ Lloret-Fillol *et al.* ^13^ and Nocera *et al.* ^21*a*^, respectively. For the Fe^IV^ intermediates, light was shown to disproportionate [{(corrole)Fe^IV^}_2_-μ-oxo] to form the corresponding [(corrole)Fe^V^(O)], hence increasing its reactivity. For the corresponding [Fe^IV^(O)(MePy_2_tacn)]^2+^, the increase in reactivity was explained by the formation of an excited state *via* spin change. The possibility of rate enhancement in the reactivity of **2** due to the presence of light clearly exists and is currently being investigated.

## Conclusions

In conclusion, we have demonstrated the first example of an iron-complex catalysed photocatalytic hydroxylation and epoxidation reaction, with a variety of substrates, using water as the oxygen source. The [{(bTAML)Fe^IV^}_2_(μ-O)]^2–^ (**2**) dimer, produced by oxidative activation of a water molecule, remains the active oxidant and results in hydroxylation or epoxidation with high selectivity. ^18^O-labelling experiments support the metal-based selective and controlled oxygenation of substrates with C–H and C=C bonds. Although the reactivity of this photochemical system is lower compared to that of the Fe^III^-bTAML/NaOCl system and the use of [Co(NH_3_)_5_Cl]^2+^ as the electron acceptor is not optimal, we believe that conjugating complex **1** to a light harvesting system can increase its efficiency manyfold. Such work is being attempted currently in our laboratory.

## Experimental section

### General procedure for the photocatalytic hydroxylation reactions

The photocatalytic oxidation of alkanes was carried out under an argon atmosphere in an acetonitrile and phosphate buffer (pH 10, 10 mM) (3 : 2 v/v) mixed solvent. The reaction solution (1.0 mL) containing catalyst **1** (1.0 × 10^–4^ M) [Ru(bpy)_3_]Cl_2_·6H_2_O (2.0 × 10^–3^ M), [Co(NH_3_)_5_Cl]Cl_2_ (2.0 × 10^–2^ M) and substrate (3.0 × 10^–3^ M) was irradiated with a blue LED light source (3 W, 440 nm) and stirred for 40 min at room temperature. The temperature was kept constant using a water circulating system during the whole reaction. The final reaction mixture was extracted with dichloromethane (five times with 2 mL of dichloromethane each time) and dried over Na_2_SO_4_. After concentrating the reaction solution by purging with nitrogen gas, the product was identified and quantified by GC-MS. Control experiments were performed under the same conditions as mentioned above.

### General procedure for the photocatalytic epoxidation reactions

The photocatalytic epoxidation of alkenes was carried out in an acetonitrile and phosphate buffer (pH 10, 10 mM) (3 : 2 v/v) mixed solvent. The reaction solution (1.0 mL) containing catalyst **1** (1.0 × 10^–4^ M), [Ru(bpy)_3_]Cl_2_·6H_2_O (2.0 × 10^–3^ M), [Co(NH_3_)_5_Cl]Cl_2_ (2.0 × 10^–2^ M) and substrate (5.0 × 10^–3^ M) was irradiated with a blue LED light source (3 W, 440 nm) and stirred for 40 min at room temperature (27 °C). The temperature was kept constant using a water circulating system during the whole reaction. After 40 min, the reaction mixture was extracted with dichloromethane (five times with 2 mL of dichloromethane each time) and dried over Na_2_SO_4_. After concentrating the reaction solution by purging with nitrogen gas, the product was identified and quantified using GC-MS.

### ^18^O-labelling experiment

^18^O-labelling experiments were carried out for the photochemical oxidation of styrene, adamantane, *cis*-1,2-dimethylcyclohexane and ambroxide. A mixture of acetonitrile and H_2_^18^O (3 : 2 v/v) solution containing catalyst **1** (1.0 × 10^–4^ M), [Ru(bpy)_3_]Cl_2_·6H_2_O (2.0 × 10^–3^ M), [Co(NH_3_)_5_Cl]Cl_2_ (2.0 × 10^–2^ M) and substrates (styrene, adamantane, *cis*-1,2-dimethylcyclohexane and ambroxide) (5.0 × 10^–3^ M) was stirred and irradiated with light (3 W blue LED, 440 nm) for 40 min at room temperature. The resulting solution was extracted with dichloromethane and the products were analyzed by GC-MS.

### UV-vis experiment

The photochemical generation of [{(bTAML)Fe^IV^}_2_-μ-oxo]^2–^ (**2**) was observed by UV-vis spectroscopy. A solution mixture of acetonitrile and phosphate buffer (3 : 2 v/v) containing catalyst (1.0 × 10^–4^ M), [Ru(bpy)_3_]Cl_2_·6H_2_O (2.0 × 10^–5^ M) and [Co(NH_3_)_5_Cl]Cl_2_ (6.0 × 10^–4^ M) was added to a 1.0 cm (path length) quartz cuvette and spectra were recorded at 0, 1 and 3 min of photoirradiation with a blue LED light source (3 W, 440 nm). The chemical formation of [{(bTAML)Fe^IV^}_2_-μ-oxo]^2–^ (**2**) was also examined from changes in the absorption spectra of the solution mixture of acetonitrile and phosphate buffer (3 : 2 v/v) containing catalyst (**1**) and NaOCl (0.5 equivalent) (Fig. S5†).

## Conflicts of interest

There are no conflicts to declare.
