# Supplementary material for: Selective photocatalytic hydroxylation and epoxidation reactions by an iron complex using water as the oxygen source
Source: Chem Sci. 2017 Sep 4;8(11):7545–51. doi: 10.1039/c7sc02780j (PMC5676249; doi:10.1039/c7sc02780j)
Supplement: Supplementary file 1 [file SC-008-C7SC02780J-s001.pdf]

Electronic Supplementary Material (ESI) for Chemical Science.
This journal is © The Royal Society of Chemistry 2017

Supporting Information

Selective Photocatalytic Hydroxylation and Epoxidation Reaction by an Iron Complex Using Water as the Oxygen Source

Bittu Chandra[#], Kundan K. Singh[#] and Sayam Sen Gupta*[a]

[#] Both authors contributed equally to this work

Table of Contents **Page No.**

Materials……………………………………………………………………………………..................S2

General Instrumentations…………………………………………………………………...............S2

Quantum yield determination………………………………………………………………………….S3

Table S1. Quantum yields of alkanes………………………………………………………………..S3

Table S2. . Quantum yields of alkenes…………………………………….………………………………S4

Figure S1. GC-MS spectra of ^{18}O-labelling experiment for *cis*-1,2-dimethylcyclohexane with $H_2{}^{18}O$……S5

Figure S2. GC-MS spectra of ^{18}O-labelling experiment for ambroxide with $H_2{}^{18}O$………………………...S6

Figure S3. TON of styrene oxide *vs*. percentage of water (effect of water) in solvent mixture …..........S7

Figure S4. ^{1}H-NMR spectra of *cis*- and *trans*-stilbene oxide formed in photochemical reaction of cis-stilbene……………………………………………………………………………………………………S8

Figure S5. UV-vis spectra of **2** synthesized with NaOCl and complex **1** ………………………............S9

Figure S6. HR-MS of complex **1** after completion of the photochemical reaction……………………......S10

References…………………………………………………………………………………………...S11

Experimental Section

Materials

All reagents were obtained commercially and used without further purification unless otherwise noted. $[(bTAML)Fe\text{-}OH_2]^-$ (**1**) (bTAML = biuret-modified tetraamidomacrocyclic ligands) was synthesized by our previously reported method.[1] Tris(2,2'-bipyridine) ruthenium(II) chloride hexahydrate $[Ru^{II}(bpy)_3]Cl_2.6H_2O$ was purchased from Sigma-Aldrich and used as received. $[Co^{III}(NH_3)_5Cl]Cl_2$ was purchased from Alfa Aesar and used as received. Acetonitrile (HPLC grade, Aldrich) was used after passing through an activated neutral alumina column. Purification of water (18.2 MΩ cm) was performed with a Milli-Q system. ^{18}O-enriched water (98%) was procured from the Shanghai Research Institute of Chemical Industry (China). All the substrates were purchased from Aldrich and TCI and were passed through activated neutral alumina and distilled prior to use.

General Instrumentations

UV−vis spectral studies were carried out using an Agilent diode array 8453 spectrophotometer with an attached electrically controlled thermostat. Gas chromatography (GC) was performed on a Perkin Elmer Arnel Clarus 500 instrument equipped with a hydrogen flame ionization detector; BP20 columns (polar) (12 m × 0.32 mm × 1.0 µm) were used with helium as the carrier gas at a flow rate of 1 mL min^{-1}. GC-MS was performed on a Thermo Scientific ISQ QD Mass Spectrometer attached with Thermo Scientific TRACE 1300 gas chromatograph using an HP-5ms capillary column (30 m × 0.25 mm × 0.25 µm, J&W Scientific) with helium as the carrier gas. HR-MS was performed in a Thermo Scientific Q-Exactive Orbitrap analyzer using an electrospray ionization source connected with a C18 column (150 m × 4.6 mm × 8.0 µm). A homemade apparatus of light source which contains an array of six blue LEDs (λ_{max} = 440 nm, 3 watt) with a metallic water circulator having six holes to hold the reaction vials exactly over the lights used for photochemical oxidations.

Quantum yield determination:

The quantum yield of the photochemical reactions were determined by using a standard actinometer (potassium ferrioxalate).[3,4] The reaction solution (1.0 mL) containing catalyst **1** (1.0×10^{-4} M), $[Ru(bpy)_3]Cl_2.6H_2O$ (2.0×10^{-3} M), $[Co(NH_3)_5Cl]Cl_2$ (2.0×10^{-2} M) and substrate (3.0×10^{-3} M for alkanes and 5.0×10^{-3} M for alkenes) was irradiated with a blue LED light source (λ_{max} = 440 nm, 3 W) for 40 min. The product formed after the photochemical reaction was quantified using GC-MS. The photon flux was calculated to be 1.95×10^{-8} einstein s^{-1}. The quantum yields after the photochemical reaction of alkanes and alkenes were calculated using the following equation:

$$\Phi = \frac{number\ of\ product\ molecules \times 2}{number\ of\ incident\ photons} \times 100\ \%$$

Table S1. Quantum yields of alkanes

**Entry**	**Substrate (Alkanes)**	**Quantum Yield (%)**
1.	Adamantane	8.6
2.	Cumene	4.3
3.	*cis*-1,2-Dimethylcyclohexane	7.0
4.	*cis*-decalin	12.2
5.	*trans*-decalin	3.8
6.	Cedryl acetate	4.6
7.	Ambroxide	7.0
8.	Diphenyl methane	7.2

Table S2. Quantum yields of alkenes

Entry	Substrate (Alkenes)	Quantum Yield (%)
1.	Styrene	13.9
2.	4-chlorostyrene	9.0
3.	4-methoxystyrene	18.7
4.	*cis*-stilbene	9.8
5.	*cis*-cyclooctene	10.2
6.	Norbornene	12.6

Reaction condition: photochemical reaction yields were calculated using **1** (1.0×10^{-4} M) $[Ru(bpy)_3]Cl_2.6H_2O$ (2.0×10^{-3} M), $[Co(NH_3)_5Cl]Cl_2$ (2.0×10^{-2} M) and substrate (3.0×10^{-3} M for alkanes and 5.0×10^{-3} M for alkenes) was irradiated with a blue LED light source (λ_{max} = 440 nm, 3 W) for 40 min. The quantum yields were calculated using the above mentioned equation.

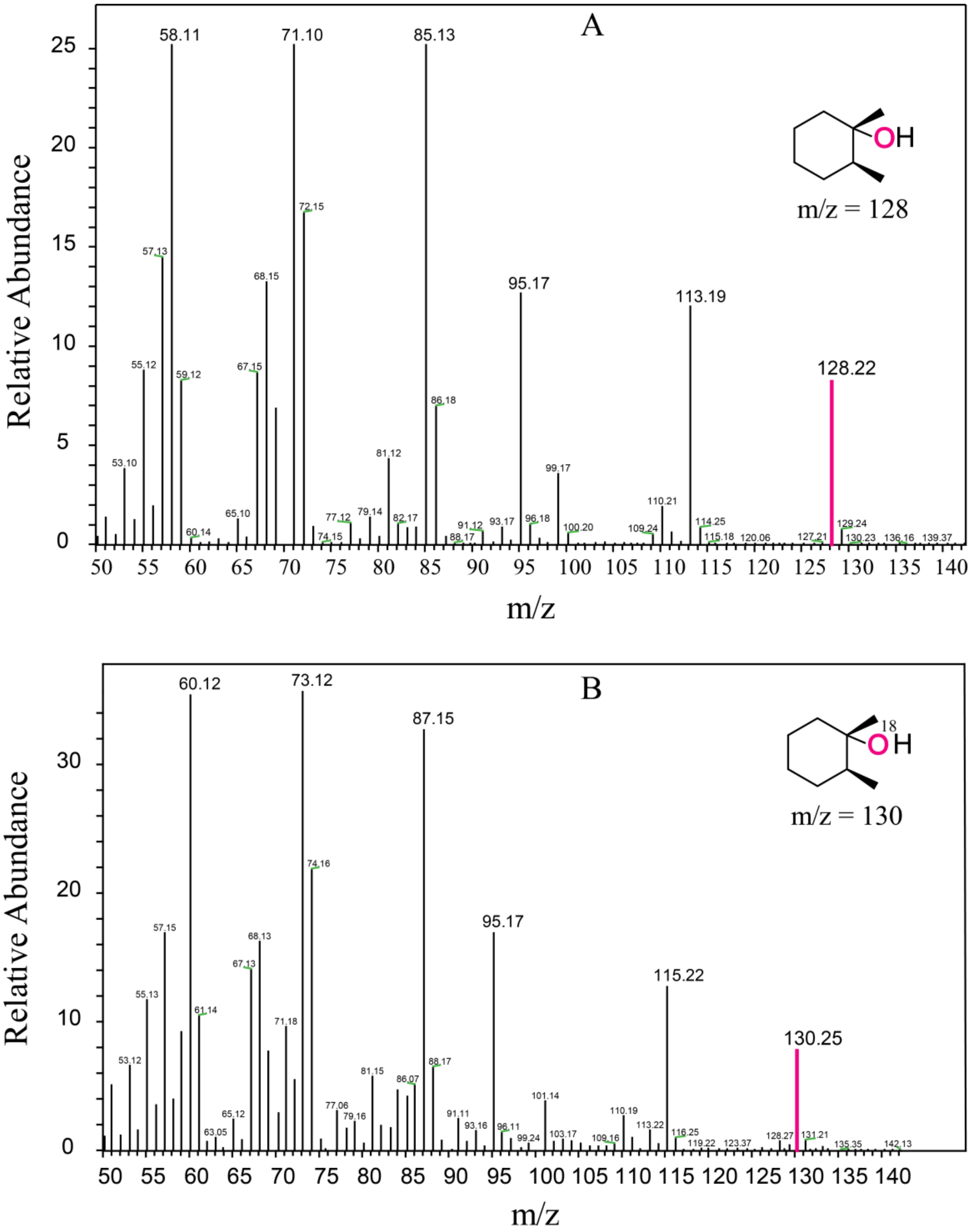

Fig. S1 GC-MS spectra of product after photochemical oxidation reaction with *cis*-1,2-dimethylcyclohexane as the substrate A) in $H_2{}^{16}O$; B) in $H_2{}^{18}O$.

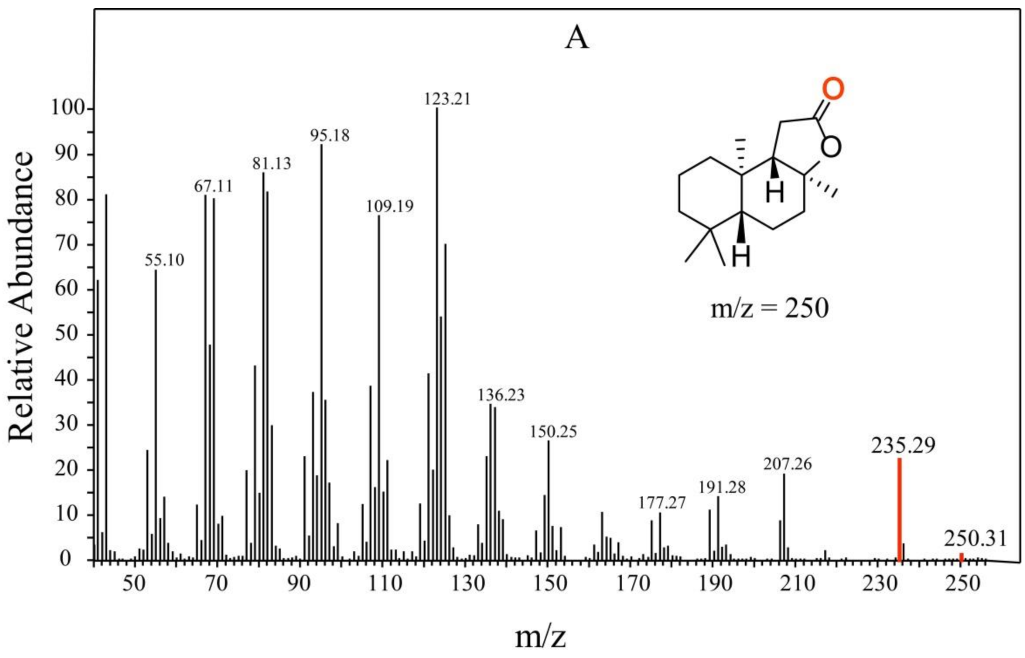

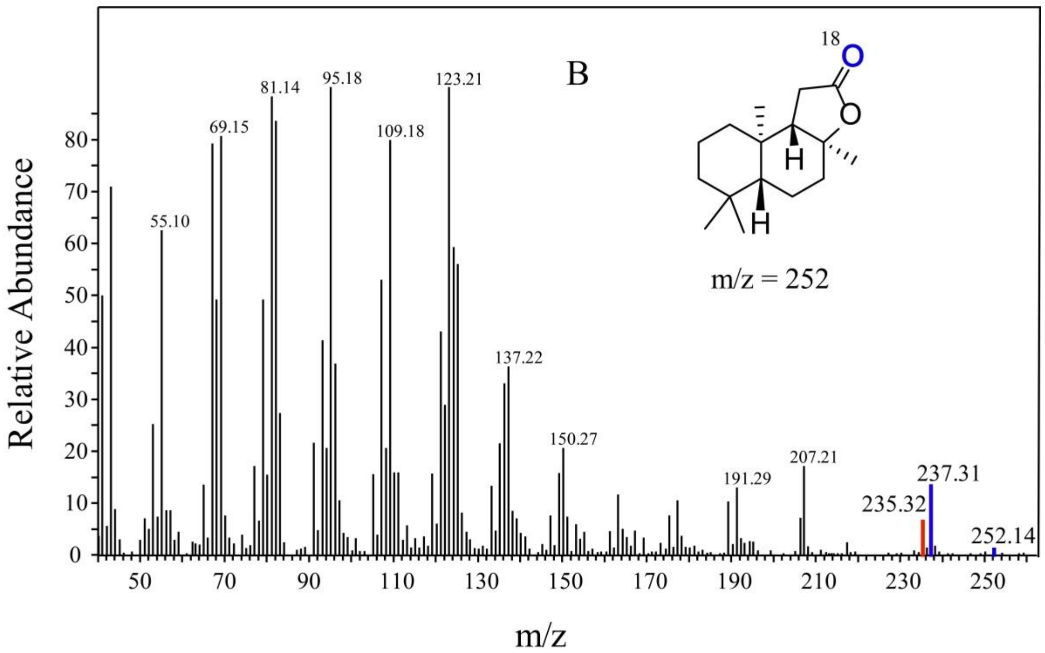

Fig. S2 GC-MS spectra of product after photochemical oxidation reaction ambroxide (30 mM) as the substrate with catalyst **1** (0.1 mM) A) in $H_2{}^{16}O$; B) in $H_2{}^{18}O$

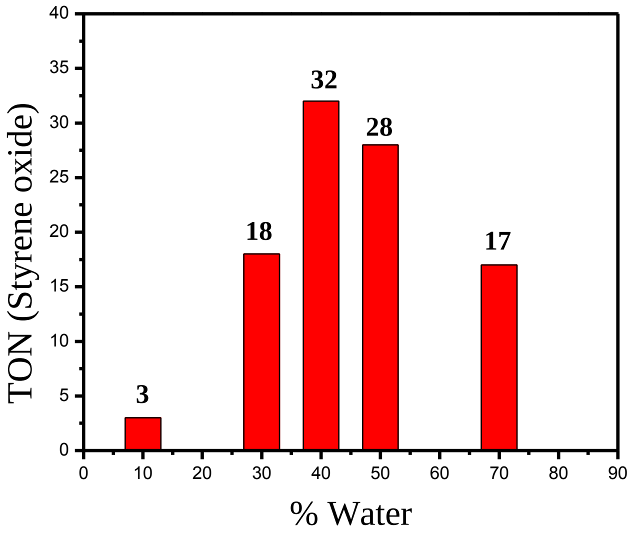

Fig. S3. Turnover number (TON) of styrene oxide *vs*. percentage of water in aqueous phosphate buffer-acetonitrile solvent mixture for the photochemical oxidation reaction of styrene with complex **1**. Using catalyst **1**, photosensitizer $[Ru^{II}(bpy)_3]^{2+}$ and sacrificial electron acceptor $[Co(NH_3)_5Cl]^{2+}$ under blue LED light irradiation, we optimized the concentration of water in acetronitrile-water (phosphate buffer) mixture for the epoxidation of styrene. A very low product yield (TON 3) in 1:9 water-CH_3CN mixtures was attributed to the poor solubility of $[Ru^{II}(bpy)_3]^{2+}$ and $[Co(NH_3)_5Cl]^{2+}$ in organic solvent (acetonitrile). When concentration of water was increased to 40%, the TON for styrene oxide increased to 32. Upon further increment of water concentration (70% water), epoxide product formation decreased to give TON of 17 which was most likely due to the insolubility of substrate as well as an enhanced competitive reaction of reactive intermediate with water.[1b,2]

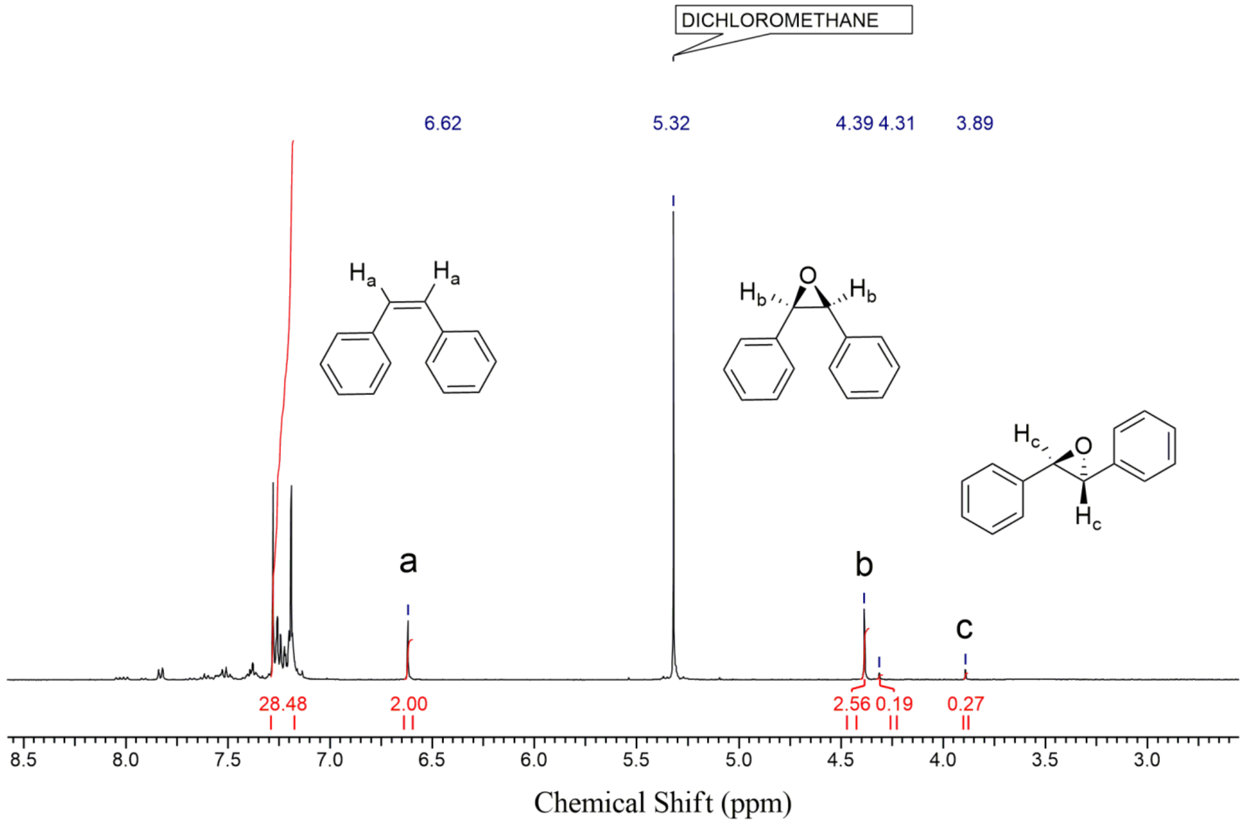

Fig. S4 ^{1}H-NMR spectra of *cis*-stilbene oxide (major) and *trans*-stilbene oxide (minor) formed in photochemical reaction of *cis*-stilbene. The ratio of *cis* to *trans* product was estimated to be 19:2.

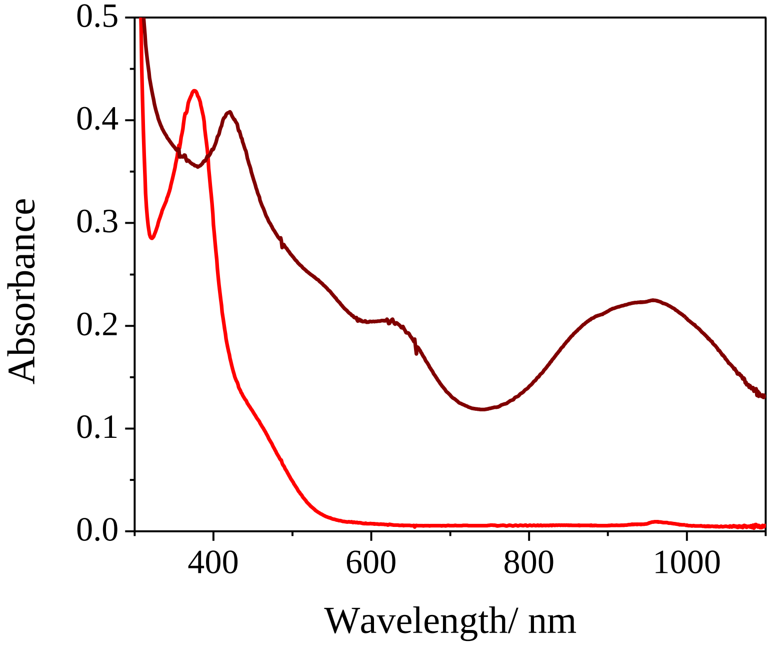

Fig. S5 UV-vis spectra of complex **2** (brown) formed with NaOCl and complex **1**(red).

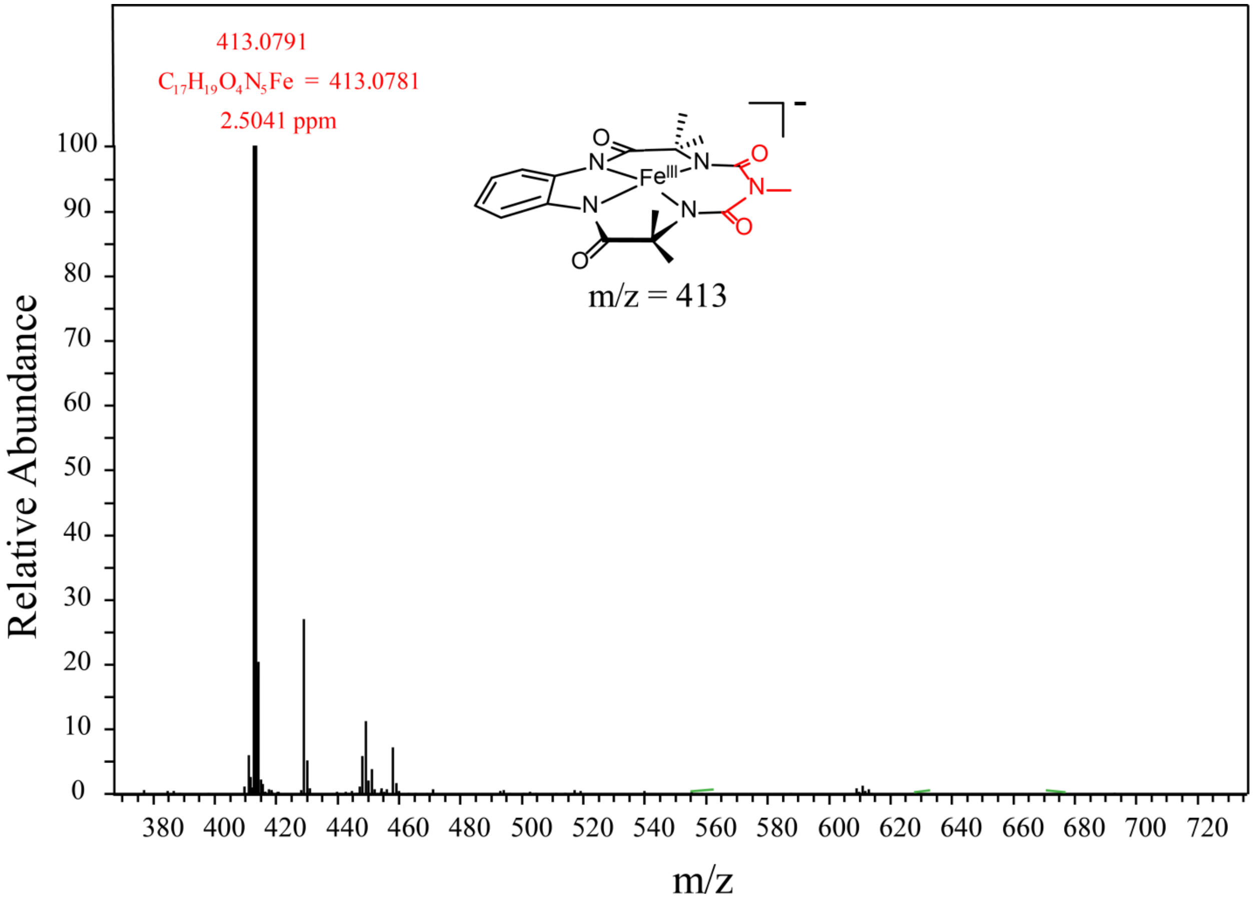

Fig. S6 HR-MS of catalyst **1** after 40 minutes of photochemical oxidation reaction with styrene using **1** (1.0×10^{-4} M), $[Ru^{II}(bpy)_3]^{2+}$ (2.0×10^{-5} M) and $[Co^{III}(NH_3)_5Cl]^{2+}$ (6.0×10^{-4} M) in acetonitrile-aqueous phosphate buffer (3:2 v/v) solvent.

References

1 (a) C. Panda, M. Ghosh, T. Panda, R. Banerjee and S. Sen Gupta, *Chem. Commun.*, 2011, **47**, 8016-8018; (b) C. Panda, J. Debgupta, D. Diaz Diaz, K. K. Singh, S. Sen Gupta and B. B. Dhar, *J. Am. Chem. Soc.*, 2014, **136**, 12273-12282.

2 K. K. Singh, M. k. Tiwari, B. B. Dhar, K. Vanka and S. Sen Gupta, *Inorg. Chem*., 2015, **54**, 6112-6121.

3 C. G. Hatchard and C. A. Parker, *Proc. R. Soc. London, Ser. A,* 1956**, 235**, 518.

4 M. N. Hopkinson, A. G.-Suárez, M. Teders, B. Sahoo and F. Glorius, *Angew. Chem. Int. Ed.* 2016, **55**, 4361-4366.
